# Pattern of Adverse Drug Reactions Associated with the Use of Anticancer Drugs in an Oncology-Based Hospital of Nepal

**DOI:** 10.31662/jmaj.2021-0015

**Published:** 2022-09-26

**Authors:** Ramisa Tamang, Laxman Bharati, Asmita Priyadarshini Khatiwada, Akihiko Ozaki, Sunil Shrestha

**Affiliations:** 1 Department of Pharmacy, Maharajgunj Medical Campus, Institute of Medicine, Tribhuvan University, Kathmandu, Nepal; 2 Department of Pharmaceutical and Health Service Research, Nepal Health Research and Innovation Foundation, Lalitpur, Nepal; 3 Medical Governance Research Institute, Tokyo, Japan; 4 Department of Breast Surgery, Jyoban Hospital of Tokiwa Foundation, Fukushima, Japan; 5 Nobel College Faculty of Health Sciences, Pokhara University, Sinamanagal, Kathmandu, Nepal

**Keywords:** Adverse drug event, Adverse drug reaction, Anticancer drugs, Chemotherapy, Nepal

## Abstract

**Introduction:**

Adverse drug reactions (ADRs) are among the leading causes of morbidity and mortality worldwide. ADRs of anticancer drugs are ubiquitous. However, in Nepal, studies on chemotherapy-induced ADRs are scarce. Thus, this study aimed to assess the ADRs associated with the use of anticancer drugs and their management along with causality assessment and severity of ADRs.

**Methods:**

A prospective cross-sectional observational and single-center study was conducted at Bhaktapur Cancer Hospital, Nepal, for 6 months. All the patients who fulfilled the study criteria were analyzed to identify ADRs occurring daily. In addition, all collected data were recorded and analyzed using descriptive statistics.

**Results:**

A total of 861 ADRs were detected among 102 cancer patients. The mean ± S.D. age of the patients was 49.93 ± 14.27 years, and each enrolled patient experienced one or more ADRs with a mean ± S.D. of 8.44 ± 3.27. The common ADRs observed were fatigue, anorexia, alopecia, constipation, nausea, vomiting, and neuropathy. Cyclophosphamide, either alone or in combination with other chemotherapeutic agents, was responsible for most ADRs. According to Naranjo’s causality assessment algorithm, most of the ADRs belonged to the probable (47.1%) category. Majority (54.9%) of the ADRs were moderate in their severity. Proton pump inhibitors, antiemetic, mouth gargle, protein powders, iron tablets, and multivitamin and mineral tablets were commonly used for ADR management.

**Conclusions:**

The occurrence of chemotherapy-related ADRs in each enrolled patient is a crucial concern. The present study highlights the need for active monitoring of the patients to identify and manage ADRs promptly.

## Introduction

Cancer is one of the most threatening diseases for humans. In 2012, an estimated 14.1 million new cancer cases and 8.2 million cancer-related deaths were observed, and the incidence of cancer is predicted to increase to 19.3 million by 2025 ^(1)^. There are different treatment approaches for cancer, such as surgery, chemotherapy, radiation therapy, immunotherapy, and monoclonal antibody therapy, with chemotherapy being commonly used as part of a multimodal treatment strategy for different cancer types ^(2)^.

A variety of antineoplastic or chemotherapeutic agents are used to treat cancer and are proven to be beneficial and considerably improve patients’ quality of life. However, the toxicity and narrow therapeutic index of these drugs ^(3)^ may extend their therapeutic effects, which can be acute or chronic, temporary or permanent, and mild or potentially life-threatening ^(4), (5)^. This makes the vigilance of drug use crucial in every cancer patient.

Adverse drug reaction (ADR) is the fifth leading cause of mortality worldwide, and it accounts for approximately 3%-4% of all hospital admissions ^(6), (7)^. Among various drugs associated with ADRs, antineoplastic agents rank first in terms of toxicity ^(8)^. Furthermore, the ADRs of anticancer drugs are intensified by the frequent use of a combination of drugs compared with single-drug therapy ^(9)^. ADRs associated with antineoplastic agents include alopecia, nausea and vomiting, myelosuppression, cardiac toxicity, hemorrhagic cystitis, mucositis, hot flushes, electrolyte imbalance, and deep vein thrombosis ^(10)^. Failure to manage these effects promptly may result in fatal outcomes, increase the healthcare cost for patients, and affect the patients’ quality of life. Hence, timely management of such serious adverse effects is crucial.

Furthermore, studies addressing the ADRs associated with chemotherapy should make the community aware of the potential ADRs. In Nepal, the burden of cancer has been increasing over time, which has increased the need for chemotherapy ^(11)^. In 2017, cancer accounted for 10% of the total deaths, and the estimates have been increasingly coming to 2020 ^(12), (13)^, with lung cancer being the leading cause of deaths among cancer cases. In addition to lung cancer, cervical, breast, stomach, and colorectal cancers are commonly observed in Nepalese ^(14)^. A study using a population-based cancer registry demonstrated that 95.7 per 1000000 population is the age-adjusted incidence rate ^(15)^. Although cancer is one of the common diseases observed in Nepal, studies ^(16), (17), (18), (19), (20), (21)^ on the ADRs of chemotherapy in oncology-based settings in Nepal are scarce. Likewise, the pharmacovigilance system of anticancer drugs is limited and underreported ^(22)^. This may be due to a lack of support from hospital management, lack of team spirit in the admission and reporting of ADRs by the healthcare professionals, and sometimes even the fear of legal implications from patients or the patients’ party ^(23), (24), (25), (26)^. Consequently, the current study was conducted to investigate the ADRs of chemotherapeutic agents in general. This study aimed to identify the pattern, frequency, causality, and severity of ADRs experienced by cancer patients taking various chemotherapeutic agents for their relevant conditions as well as to assess the management approaches for the ADRs that occurred.

## Materials and Methods

### Study design and study site

A prospective cross-sectional observational study was conducted over 6 months from August 2018 to February 2019 at Bhaktapur Cancer Hospital, Bhaktapur, Nepal. It is a 110-bed national-level cancer hospital in Nepal that provides chemotherapy, radiotherapy, surgery, and brachytherapy, including palliative care.

### Study sample

A total of 102 cancer patients were enrolled in this study. The non-probability sampling method (purposive sampling) was employed to determine the sample of the study population. Patients above 18 years old diagnosed with cancer and were on active treatment with chemotherapy were included in this study. Pregnant women and patients being treated with radiotherapy were excluded from this study.

### Ethical approval

The study was approved by the Institutional Review Board of the Institute of Medicine, Tribhuvan University Teaching Hospital, Kathmandu, Nepal. The permission to conduct the study was given by Bhaktapur Cancer Hospital, Bhaktapur, Nepal. The patients who fulfilled the study criteria were enrolled after written and verbal informed consent was obtained from them and/or their caregivers.

### Data collection tools and techniques

The clinical and demographic data of the participants relevant for the study were obtained from their medical records and documented in the suitably designed data collection form (Annexure 1). All the required details of the suspected ADRs were collected and documented in the same form. The causality of the ADRs was evaluated using Naranjo’s algorithm ^(27)^, and severity was assessed using the Modified Hartwig and Siegel scale ^(28)^.

### Data analysis

Descriptive statistics such as percentage, mean, and standard deviations were used to describe the study variables. The analysis was conducted using the SPSS software for Windows, version 21.0 (IBM Corp., Armonk, NY).

## Results

A total of 102 patients who fulfilled the inclusion criteria were enrolled in this study. The patients’ average age was 49.93 ± 14.3 years, and majority (n = 45, 44.1%) of them belonged to the age group of 45-59 years. The ADR distribution was higher in women (n = 74, 72.5%). Majority (n = 28, 27.5%) of the enrolled patients were found to have stage II cancer, and most of them (n = 28, 27.5%) were undergoing the third cycle of chemotherapy. Furthermore, a total of 24 different cancer types were detected in the study, with breast cancer having the highest frequency (n = 35, 34.3%). The detailed demographic, disease, and clinical characteristics of the patients are presented in Table 1.

**Table 1.** Demographic and Clinical Characteristics of the Study Population.

Characteristics	Group	Frequency (%) N = 102
Age: Mean ± SD	49.93 ± 14.27	
Age group (years)	15-29	10 (9.8)
	30-44	18 (17.6)
	45-59	45 (44.1)
	60-74	25 (24.5)
	>75	4 (3.9)
Sex	Female	74 (72.5)
	Male	28 (27.5)
Occupation	Farmer	39 (38.2)
	Housewife	31 (30.4)
	Business	12 (11.8)
	Student	5 (4.9)
	Service	9 (8.8)
	Others	6 (5.9)
Marital status	Single	93 (91.2)
	Married	7 (6.9)
	Divorced	2 (2)
**Cancer type**	Breast cancer	35 (34.3)
	Lung cancer	15(14.7)
	Ovarian cancer	8 (7.8)
	Stomach cancer	6 (5.9)
	Rectal cancer	6 (5.9)
	Cervical cancer	4 (3.9)
	Pancreatic Cancer	3 (2.9)
	Immature teratoma	3 (2.9)
	Urinary bladder cancer	3 (2.9)
	Cholangiocarcinoma	2 (1.9)
	Gallbladder Cancer	2 (1.9)
	Choriocarcinoma	2 (1.9)
	Non-Hodgkin lymphoma (NHL)	2 (1.9)
	Colon Cancer	1 (0.9)
	Prostate Cancer	1 (0.9)
	Acute lymphoblastic leukemia (ALL)	1 (0.9)
	Acute myeloid leukemia (AML)	1 (0.9)
	Hodgkin’s Lymphoma	1 (0.9)
	Vulval Cancer	1 (0.9)
	Oesophageal cancer	1 (0.9)
	Gestational Trophoblastic Neoplasia (GTN)	1 (0.9)
	Periampullary cancer	1 (0.9)
	Rectosigmoid cancer	1 (0.9)
	Endometrial cancer	1 (0.9)
Stage of cancer	I	7 (6.9)
	II	28 (27.5)
	III	25 (24.5)
	IV	25 (24.5)
	Stage not defined	17 (16.6)
Chemotherapy cycle	Second	19 (18.6)
	Third	28 (27.5)
	Fourth	14 (13.7)
	Fifth	20 (19.6)
	Sixth	12 (11.8)
	Seventh	3 (2.9)
	Ninth	4 (3.9)
	Others	2 (2)

Of the 233 anticancer drugs prescribed for 102 patients in the study, alkylating agents were most frequently prescribed (n = 114, 48.92%). Cyclophosphamide (n = 39, 16.7%), followed by carboplatin (n = 34, 14.6%) and then cisplatin (n = 34, 14.6%), was found to be the widely used alkylating agent. Other classes of anticancer drugs prescribed are listed in Supplementary Table 1.

ADRs were observed in all patients enrolled in the current study, and the mean of the ADRs that occurred was 8.44 ± 3.3. Thus, a total of 861 ADRs were detected in the study. Patients with breast cancer (n = 304, 35.3%) had the highest frequency of ADRs, followed by those with lung cancer (n = 100, 11.6%) and then those with ovarian cancer (n = 65, 7.5%) (Table 2).

**Table 2.** Types of Cancer and ADR Distribution According to Cancer Type.

Cancer type	ADR frequency (%) (N=861)
Breast cancer	304 (35.3)
Lung cancer	100 (11.6)
Ovarian cancer	65 (7.5)
Stomach cancer	52 (6.0)
Rectal cancer	51 (5.9)
Cervical cancer	35 (4.0)
Pancreatic cancer	15 (1.7)
Urinary bladder cancer	26 (3.0)
Immature teratoma	26 (3.0)
Non-Hodgkin lymphoma (NHL)	21 (2.4)
Cholangiocarcinoma	22 (2.5)
Others*	144 (16.8)

*Others include prostate cancer, esophagus, vulva, GTN, peri ampulla, endometrium, cholangiocarcinoma, and colon.

Among 102 patients included in this study, 861 ADRs were recorded, with the most common being fatigue (n = 86, 84.3%), followed by anorexia (n = 81, 79.4%) and alopecia (n = 65, 63.7%). The common hematological reactions were anemia (40.2%) and neutropenia (41.1%). Other adverse reactions are listed in Table 3.

**Table 3.** Pattern of ADRs Observed.

Pattern of Adverse drug reactions	Frequency (%) (N=102)
Fatigue	86 (84.3)
Anorexia	81 (79.4)
Alopecia	65 (63.7)
Constipation	44 (43.1)
Nausea	43 (42.1)
Neutropenia	42 (41.1)
Anemia	41 (40.2)
Vomiting	40 (39.2)
Neuropathy	37 (36.2)
Injection site reaction	32 (31.3)
Nail changes	27 (26.4)
Malaise	26 (25.4)
Edema	23 (22.5)
Taste changes	14 (13.7)
Weight changes	16 (15.6)
Diarrhea	17 (16.6)
Fever	19 (18.6)
Hyperpigmentation	14 (13.7)
Dyspepsia	13 (12.7)
Febrile neutropenia	10 (9.8)
Blood pressure changes	9 (8.8)
Burning micturition	9 (8.8)
Dyspnea	9 (8.8)
Thrombocytopenia	8 (7.8)
Irregular menses	7 (6.8)
Joint pain	7 (6.8)
Insomnia	7 (6.8)
Anaphylaxis	5 (4.9)
Difficulty swallowing	5 (4.9)
Infection	6 (5.8)
Irritability	5 (4.9)
Flatulence	5 (4.9)
Cough	4 (3.9)
Eye problems	2 (1.9)

ADRs of adjuvant chemotherapy (n = 62, 60.8%) were frequently observed, followed by those of palliative care (n = 27, 26.5%) and then curative care (n = 13, 12.7%). Cyclophosphamide was responsible for the occurrence of the majority (n=347, 40.3%) of the ADRs, followed by 5-FU (n=284, 32.9%) and carboplatin (n=266, 30.9%). The list of drugs associated with the observed ADRs is provided in Table 4.

**Table 4.** Drugs Responsible for Causing ADRs (Alone or in Combination).

Anticancer drugs	Frequency (%) (N=861)
Cyclophosphamide	347 (40.3)
5-fluorouracil	284 (32.9)
Carboplatin	266 (30.9)
Gemcitabine	177 (20.5)
Docetaxel	176 (20.4)
Doxorubicin	166 (19.2)
Epirubicin	145 (16.8)
Cisplatin	115 (13.3)
Etoposide	92 (10.6)
Paclitaxel	88 (10.2)
Oxaliplatin	60 (6.9)
Vincristine	54 (6.2)
Methotrexate	53 (6.1)
Bleomycin	44 (5.1)
Actinomycin	36 (4.1)
Vinblastine	18 (2.0)
Others	44 (5.1)

*Others: cytarabine, trastuzumab, irinotecan, bicalutamide, pemetrexed, decitabine

The most affected organ system was the gastrointestinal (GI) organs (94.1%), followed by the skin (75.4%) and hematological organs (67.6%). Details of the affected system organ class are summarized in Supplementary Table 2.

**Figure 1.**
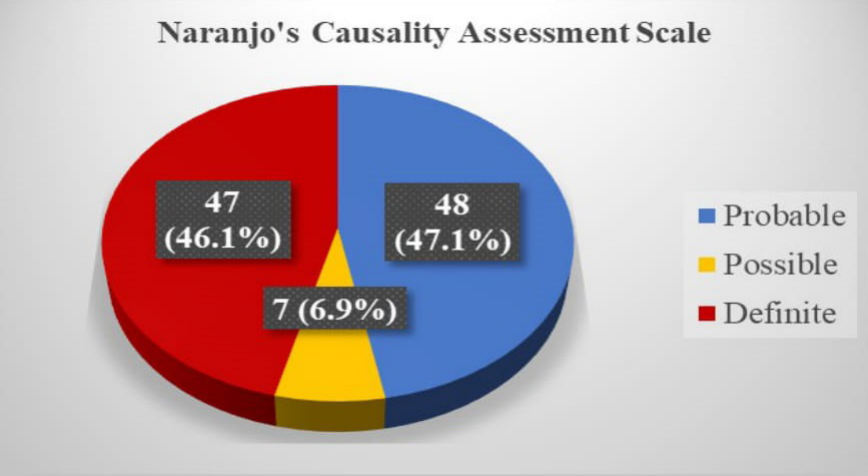
Naranjo’s Causality Assessment of observed ADRs in the patients.

Most of the ADRs belonged to the “probable” category (47.1%), followed by “possible” (46.1%), as assessed using Naranjo’s causality assessment algorithm (Figure 1).

**Figure 2.**
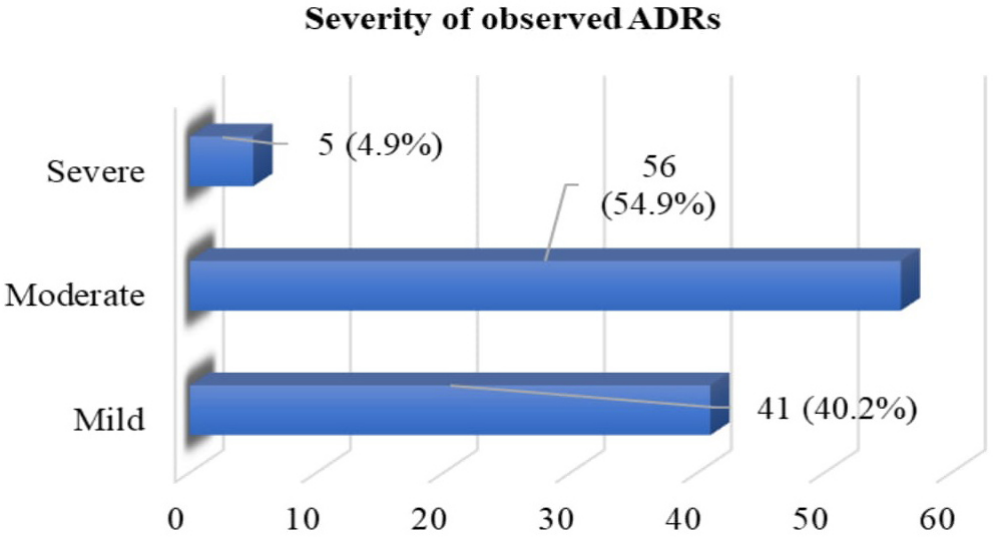
Modified Hartwig and Siegel Scale of observed ADRs in the patients.

The assessment of the severity of the observed ADRs using the Modified Hartwig and Siegel scale indicated that 54.9% and 40.2% of the ADRs were moderate and mild, respectively (Figure 2).

All the study subjects (n = 102, 100%) were prescribed proton pump inhibitors (PPIs), intravenous (IV) regular saline flush, and antiemetics to manage the ADRs of various chemotherapeutic agents. The different therapeutic classes prescribed to manage the reported ADRs are listed in Table 5.

**Table 5.** Therapeutic Class Used in ADRs Management.

Class	Frequency (%) N=102
Proton Pump Inhibitors	102 (100.0)
IV Normal Saline flush	102 (100.0)
Anti-emetics	102 (100.0)
Protein powder	89 (87.2)
Mouth Gargle	78 (76.4)
Iron tablets	72 (70.5)
Inj. Granulocyte colony-stimulating factor	68 (66.6)
Multivitamin mineral tablets	57 (55.8)
Analgesics	34 (33.3)
Antimicrobial agents	27 (26.4)
Laxatives	25 (24.5)
Corticosteroids	24 (23.5)
Appetite stimulant	23 (22.5)
IV mannitol, Potassium Chloride, Magnesium Sulphate	15 (14.7)
Probiotics	13 (12.7)
Folinic acid	13 (12.7)
Pregabalin and Vitamin B-12	10 (9.8)
Antacid suspension	7 (6.8)
Antispasmodic	6 (5.8)
Antipsychotic	5 (4.9)
Anti-diarrheal agents	4 (3.9)
Antihistamines	2 (1.9)

The drugs used to manage various ADRs, such as nausea and vomiting, infections, hematological ADRs, and pain, are presented in Supplementary Table 3.

## Discussion

In the current study, the ADRs of anticancer drugs were observed more in women (n = 74, 72.5%) than in men, a finding consistent with those of other studies conducted in Nepal ^(17), (19), (20)^ and its neighboring countries, namely, India ^(29)^ and Bangladesh ^(30)^. This might be due to the difference in the pharmacokinetic and pharmacodynamic parameters of the drugs between women and men in general, considering the smaller body surface areas of women ^(31)^. In addition, the higher number of female patients visiting the hospital during the study period might have led to this result. However, limited studies have demonstrated higher distribution among men ^(18), (32)^ and no differences in the distribution between men and women ^(33)^.

The mean age of the patients was 49.93 years, with most of the observed ADRs occurring among patients in the age group of 45-59 years (n = 45, 44.4%), a finding similar to those of other few studies ^(17), (30), (32)^. The higher incidence of ADRs among older adults may be due to their declining metabolic capacity and excretory function, which leads to the accumulation of the drug in the body resulting in higher adverse outcomes ^(34)^.

The study population mainly consisted of farmers (n = 39, 38.2%), followed by housewives (n = 31, 30.4%), and the result was in agreement with that of another study conducted in Nepal ^(17)^. Farmers may contact a variety of substances such as pesticides, dust, oils and fumes, microbes, and zoonotic viruses, which are suspected to be carcinogens, making them more prone to cancer ^(35), (36)^.

Patients with stage II cancer (n = 28, 27.5%) were frequently observed, and most of them were undergoing the third cycle of chemotherapy. All the patients enrolled in the study experienced ADRs. Overall, the patients in our study experienced ADRs with a mean of 8.44 ± 3.3, whereas in a study conducted by Singh et al., ADRs with a mean of 4.71 ± 2.5 were observed ^(37)^. The ADRs may have been induced by the tumor itself and the treatment variables. Polychemotherapy and aging can be considered a few reasons for the increased risk of toxicity. With aging, the bone marrow reserve decreases, which increases the risk of myelosuppression complications caused by chemotherapy ^(2), (34)^.

Breast cancer was found to be common among the patients (n = 35, 34.3%), and those with breast cancer (n = 304, 35.3%) had a higher number of ADRs, a finding consistent with those of other studies ^(29), (30), (38)^. These findings are interrelated, and the higher number of women in the study supports this result.

Majority of the patients were prescribed alkylating agents, with cyclophosphamide, carboplatin, and cisplatin being the frequently prescribed ones. Cyclophosphamide (n = 347, 40.3%) was mainly responsible for ADR development, whereas other studies demonstrated that cisplatin is the primary drug that induces ADRs ^(18), (29), (32), (38), (39)^. Likewise, antimetabolites and alkylating agents were mainly associated with ADRs in a study conducted by Poddar et al ^(30)^. Cyclophosphamide is indicated for different malignant conditions, such as Hodgkin’s disease, multiple myeloma, leukemias, breast carcinoma, and adenocarcinoma of the ovary. Some of these conditions were observed in the current study, with breast cancer being the most frequently observed, thus making cyclophosphamide the most prescribed chemotherapeutic agent ^(40)^.

In the current study, the most common ADR of anticancer drugs was fatigue, followed by anorexia and alopecia. On the contrary, Poddar et al. reported nausea and vomiting as the common ADRs ^(30)^. In other studies, neutropenia and constipation were the commonly observed ADRs ^(18), (41)^. Fatigue, a common side effect of chemotherapy, might have resulted from anemia or might have been the outcome of more energy utilization by the body to combat the effects of drugs, the building of new cells, or the disposing of dead cells. Also, factors like poor appetite, emotional stress, pain, and lack of sufficient rest might have led to the condition ^(42)^.

Among the system organ classes involved, the GI system (n = 96, 94.1%) was affected mainly by ADRs, a finding similar to those of other studies ^(29), (38), (43)^. However, other studies conducted in Nepal demonstrated that the hematological system was mainly affected by the GI system ^(18), (44)^. In a single-center study, cyclophosphamide is more associated with GI side effects ^(45)^. In the current study, the higher number of cyclophosphamide prescription might have led to more GI side effects.

In this study, an assessment of the causality of ADRs using Naranjo’s algorithm indicated that the majority (n = 48, 47.1%) of the ADRs belonged to the “probable” category, followed by the “definite” category (n = 47, 46.1%), a finding that was in contrast to the results of Chopra et al.’s studies, in which most of the ADRs were under the possible (80%) or probable (20%) category ^(38)^. Most of the reactions in the current study were moderate (54.9%), followed by mild (40.2%), a finding that contrasts those of Gunaseelan et al., who reported 74.1% of the ADRs as moderate, followed by 17.9% as mild ^(46)^. Furthermore, in a study by Sharma et al., most reactions were mild to moderate and did not require withdrawal or a change in drug therapy ^(47)^.

PPIs, IV normal saline flush, and antiemetics were prescribed to all the patients in the current study. The prescription of PPIs for all patients can be justified by the high number of ADRs affecting the GI system. Because patients with cancer are prescribed multiple medications, PPIs are often used as preventive medications for possible gastritis and GI disturbances. However, the use of PPIs needs to be limited to necessary conditions because PPIs interact with and may affect the safety and efficacy of anticancer agents, such as methotrexate, capecitabine, palbociclib, enzalutamide, and anastrozole ^(48)^. Also, long-term use of PPIs increases the risk of *clostridium difficile* and other enteric infections, community-acquired pneumonia, and nutritional deficiencies ^(49)^. For every patient undergoing chemotherapy, antiemetics are compulsorily prescribed as prophylaxis; patients who experience nausea or vomiting after or during therapy need to use antiemetics. In addition, IV saline can be given to patients as supportive therapy for diarrhea, dehydration, fatigue, anorexia, and hypotension, thus rationalizing the use of IV saline flush in every patient in the study.

All the patients taking cisplatin (n = 15, 14.7%) were treated with IV fluids such as mannitol 20%, injection potassium chloride, injection magnesium sulfate to prevent nephrotoxicity, and parenteral dexamethasone, ranitidine, and granisetron were given to prevent chemotherapy-induced vomiting. For late-stage emesis, an oral antiemetic was given. In a study conducted by Saini et al., granisetron and ondansetron were prescribed to manage nausea with or without vomiting during the chemotherapy cycles ^(50)^. Furthermore, drugs like pheniramine maleate and hydrocortisone were used to manage ADRs, such as restlessness, breathlessness, and rash ^(50)^.

Considering all these findings, the authors highlight the need for an effective pharmacovigilance system in Nepal due to the absence of adequate data on adverse effects within the country and the genetic diversity of the Nepalese population. Therefore, the pharmacovigilance system in Nepal should be strengthened ^(51), (52)^. The authors also stress the urgent need for clinical pharmacy services from the clinical pharmacist for cancer patients, which will help in the monitoring and management of the ADRs, providing drug or disease-related information to cancer patients, preventing drug-drug interactions, and bridging the gap between patients and providers ^(53), (54), (55), (56)^.

### Limitations of the study

Because this is a single-center study, the findings may not be generalized, and the results may not reflect the bigger picture. In addition, the patients’ were not followed up due to time constraints, which might have led to a lack of more comprehensive information. Also, in the study, pairwise drug-ADR information was not determined, thus limiting the understanding about drugs that may be responsible for the occurrence of ADRs.

### Conclusion

Chemotherapy-related ADRs are common worldwide, most occurring among the elderly, patients with breast cancer, and patients undergoing cyclophosphamide treatment. The high incidence of these ADRs is a matter of concern. The present study highlights the need for active monitoring of the patients to identify and manage ADRs on time to ensure patient safety. Future interventional studies focused on additional data about the risk factors, including a large cohort, may help promptly detect patients at risk for ADRs and effectively manage the condition.

## Article Information

### Conflicts of Interest

Dr Akihiko Ozaki reports personal fees from MNES Inc. outside the submitted work.

### Acknowledgement

The authors would like to acknowledge Dr. Bal Mukunda Regmi, Head, Department of Pharmacy, Maharajgunj Medical Campus, Institute of Medicine, Maharajgunj, Kathmandu. The authors would also like to thank all the healthcare professionals of in-patient and daycare units of Bhaktapur Cancer Hospital, Bhaktapur and all the patients and patients’ parties who participated in this study.

### Author Contributions

RT designed the concept and design acquisition of data and interpretation of data. LB supervised the study by RT for the thesis, made substantial contributions to conception and design, and took part in revising the manuscript critically for important intellectual content. APK and SS made substantial contributions to conception and design, wrote the manuscript’s first draft, and substantially contributed to data interpretation. AO critically revised the manuscript for important intellectual content. All authors agreed to submit to the current journal, gave final approval of the manuscript, and agreed to be accountable for all aspects of the work.

### Approval by Institutional Review Board (IRB)

Research Department, Maharajgunj Medical Campus, Institute of Medicine (IOM), Tribhuvan University, Kathmandu Approval code issued by Institutional Review Board: 195(6-11-E)^2^/075/076. An approval to conduct the study was obtained from the Institutional Review Board (IRB) of the Institute of Medicine, Tribhuvan University Teaching Hospital (TUTH), Kathmandu, Nepal. The permission to conduct the study was given by Bhaktapur Cancer Hospital, Bhaktapur, Nepal. Both verbal and written informed consent was obtained from all the enrolled patients and/or their caregivers.

## Supplement

Supplementary File
