# Supplementary material for: Pattern of Adverse Drug Reactions Associated with the Use of Anticancer Drugs in an Oncology-Based Hospital of Nepal
Source: JMA J. 2022 Sep 26;5(4):416–26. doi: 10.31662/jmaj.2021-0015 (PMC9646287; doi:10.31662/jmaj.2021-0015)
Supplement: Supplementary file 1 — Supplementary File [file 2433-3298-5-4-0416-s001.pdf]

Supplementary Table 1 Different categories of anticancer drugs

Categories	Anticancer agents	Frequency (%) (N=233)
Anti-metabolites (n=59, 25.32%)	5- Fluorouracil	32 (13.7)
	Methotrexate	5 (2.1)
	Gemcitabine	22 (9.4)
Anti-cancer Antibiotics (n=8, 3.43 %)	Actinomycin	3 (1.2)
	Bleomycin	5 (2.1)
Alkylating agents (n=114, 48.92 %)	Cyclophosphamide	39 (16.7)
	Carboplatin	34 (14.6)
	Cisplatin	34 (14.6)
	Oxaliplatin	7 (3.0)
Microtubule damaging agents (n=35, 15.02 %)	Vincristine	5 (2.1)
	Vinblastine	2 (0.8)
	Docetaxel	17 (7.2)
	Paclitaxel	11 (4.7)
Topoisomerase inhibitors (n=11, 4.72 %)	Etoposide	11 (4.7)
Other different class of drugs (n=6, 2.57 %)	Cytarabine, Bicalutamide, Decitabine, Pemetrexed, Transtuzumab, Irinotecan	6 (2.5)

Supplementary Table 2 System organ class affected by observed ADRs

System organ class	ADR distribution in percent<br>Frequency (%)<br>N=102
Gastrointestinal systems	96(94.1)
Skin	77(75.4)
Hematological system	69(67.6)
Neurological system	38(37.2)
Musculoskeletal system	31(30.3)
Lymphatics	23(22.5)
Metabolic system	12(11.7)
Genitourinary system	15(14.7)
Respiratory system	14(13.7)
Cardiac System	9(8.8)
Immune system	8(7.8)

Supplementary Table 3 Drugs used for the management of the various condition

**Supportive therapy**	**Frequency (%) N=102**
Folinic acid	13(12.7)
Zoledronic acid	4(3.9)
**Drugs used for the management of nausea and vomiting**	
Inj. Dexamethasone	97(95.1)
Domperidone	56(54.9)
Ondansetron	51(50.0)
Metoclopramide	18(17.6)
Olanzapine	16(15.7 %)
**Drugs used for management of infection**	
Amikacin	4(3.9)
Cephalosporin	11(10.8)
Levofloxacin	6(5.9)
Amoxicillin and clavulanic acid	11(10.8)
Metronidazole	7(6.9)
Acyclovir	2(2.0)
Cotrimoxazole and fluconazole	2(2.0)
Tazobactam and piperacillin	2(2.0)
Co-trimoxazole	4(3.9)
**Drugs used for hematological management**	
CSF	68 (66.7)

Blood product transfusion	21 (20.5)
Hematinic (folic acid and iron)	74 (72.5)
**Drugs used for pain management**	
Paracetamol and ibuprofen (Combination)	16 (15.7)
Paracetamol	14(13.7)
Tramadol	12(11.8)
Codeine phosphate	2 (2.0)
Morphine	1 (1.0)
